# In vitro assessment of PEG-6000 coated-ZnO nanoparticles: modulating action to the resisted antibiotic activity against APEC

**DOI:** 10.1186/s12917-022-03562-4

**Published:** 2023-01-04

**Authors:** Aml Badry, Awad Abd El Hafez Ibrahim, Mohamed I. Said, Asmaa A. E. Nasr, Moemen A. Mohamed, Ahmed K. Hassan, Marwa M. Safwat

**Affiliations:** 1 Veterinarian, Avian and Rabbit Medicine Department, Faculty of Veterinary Medicine, Assiut University, Asyut, Egypt; 2 Avian and Rabbit Medicine Department, Faculty of Veterinary Medicine, Assiut University, Asyut, Egypt; 3 Department of Chemistry, Faculty of Science, Assiut University, Asyut, Egypt; 4 Poultry Diseases Department, Agriculture Research Center, Animal Health Research Institute, Assiut Lab. Egypt, Asyut, Egypt

**Keywords:** APEC, Resistance, PEG-6000 coated ZnO nanoparticles, Florfenicol, Streptomycin, MIC, FICI

## Abstract

**Background:**

Avian pathogenic *Escherichia coli* (APEC) are considered a growing health problem to both poultry and the public, particularly due to its multi-drug resistance. Zinc oxide nanoparticles (ZnO-NPs) are a promising multi-benefit candidate. This study focused on boosting the antimicrobial effect of the chemically synthesized ZnO-NPs using Polyethylene glycol-6000 (PEG-6000) and evaluating their potential to recover the sensitivity of Florfenicol and Streptomycin-resistant APEC to these drugs in a concentration range of 0.1–0.4 mg/mL. Four samples of ZnO-NPs were formulated and tested microbiologically.

**Results:**

The physicochemical characterization showed well-crystallized spherical in situ synthesized ZnO-NPs using PEG-6000 (surfactant) and ethanol (co-surfactant) of ∼19–67 nm particle size after coating with PEG-6000 molecules. These ZnO-NPs demonstrated a strong concentration-dependent antibacterial effect against multidrug-resistant APEC strains, with a minimum inhibitory concentration of 0.1 mg/mL, Combining PEG-6000 coated in situ synthesized ZnO-NPs and Florfenicol induced 60% high sensitivity (30 mm inhibitory-zone), 30% intermediate sensitivity, and 10% resistance against APEC strains. The combination with Streptomycin revealed 50% high sensitivity, 30% intermediate sensitivity, and 20% resistance with a 20 mm maximum zone of inhibition using agar well diffusion test.

**Conclusion:**

In situ preparation of ZnO-NPs using PEG-6000 and ethanol followed by coating with PEG-6000 enhanced its antibacterial activity in minimum inhibitory concentration and regained the efficacy of Florfenicol and Streptomycin against APEC, referring to a non-antibiotic antimicrobial alternative and an effective combination regimen against multidrug-resistant APEC *E. coli* in veterinary medicine.

## Background

Avian pathogenic *Escherichia coli* (APEC) are certain *E. coli* strains that have specific virulence factors, principally associated with extra-intestinal infections, mostly inducing respiratory and fatal systemic problems [1]. Antibacterial resistance is global, multifaceted phenomenon, common among APEC strains, and forms a major economic burden [2]. Multiple antimicrobial resistances and the laborious development of novel antimicrobial agents have limited the options for effective antimicrobial therapy [3]. Most recent studies have called for the necessity of retaining the activity of the available antimicrobials or searching for effective alternatives. Regaining the activity of resistant antibiotics is a challenging goal, particularly after the advent of nano-science. Owing to their effectiveness in small doses, minimal toxicity, and lack of side effects, many metal nanoparticles as ZnO-NPs were directed as antimicrobial agents [4]. ZnO-NPs are multifunctional inorganic nanoparticles that have many features like chemical and physical stability, and effective antibacterial activity [5]. The small particle size and high surface area of ZnO-NPs can enhance antimicrobial activity, causing an improvement in surface reactivity. Moreover, ZnO-NPs have selective toxicity and so are considered safe for humans and animals [6, 7]. However, the antibacterial mechanisms and underlying roles of ZnO-NPs towards bacteria are still debated.

ZnO-NPs could be synthesized by various physicochemical and biological methods. Chemically, the sol-gel process, micelle, chemical precipitation, hydrothermal method, pyrolysis, chemical vapor deposition, etc. were designed for ZnO-NPs synthesis [8].

The chemical precipitation method depends on the controlled release of Zn^2+^ cations in a homogenous solution for adjusting the kinetics of nucleation and particle growth [9].

ZnO-NPs have variable morphological forms, including nanowire, nano-flower, nano-combs, and nano-spherical shapes. The existence of water molecules around ZnO-NPs stimulates the creation of Zn-O-Zn bonds among nanoparticles, resulting in hard agglomerates (flocculation), which impede the dispersibility and applications of ZnO-NPs nanoparticles [10].

Polyethylene glycol (PEG) macromolecules adsorb to the surface of ZnO-NPs via their hydroxyl (−OH) group and hydrogen bonding interaction [11]. The PEGylation process improves the stability of suspensions against sedimentation by preventing particles from sticking together. It is proven that the PEGylation process effectively reduces the cytotoxicity of ZnO-NPs. For these reasons, this study investigated the effect of *in-*and ex-situ PEGylation on the re-dispersion of chemically synthesized ZnO-NPs in liquid media. As well, the in vitro antibacterial activity of the prepared ZnO-NPs nanoparticles and its ability to regain the activity of florfenicol and streptomycin against the resistant APEC was estimated [12].

### Materials

Zinc sulfate heptahydrate (ZnSO_4._7H_2_O), Sodium Hydroxide (NaOH), and Polyethylene glycol6000 (PEG-6000), (El-Gomhouria Co. for Trading Drugs, Chemicals & Medical Supplies, Egypt), were used. Analytical grade absolute ethanol was obtained from El-Nasr Pharmaceutical Chemical Co., Egypt.

## Methods

### Synthesis of ZnO-NPs

#### Sample 1

Bare ZnO-NPs were synthesized by the “controlled homogeneous precipitation” method according to [9, 13] with some modifications. In brief, a sodium hydroxide solution was added slowly drop-wise to an aqueous solution of zinc sulfate heptahydrate in a molar ratio of 1:0.5 under vigorous stirring for 24 hrs. A white precipitate was obtained, which was separated by centrifugation at 10,000 rpm for 5 min, washed 3 times, oven-dried at 100^o^ C, and milled to fine powders. The finally obtained powder was calcined at 500^o^ C for 2 hr. in a muffle furnace.

#### Sample 2

ZnO-NPs were in situ synthesized by using PEG-6000 as a stabilizer and ethanol as a co-surfactant. Approximately 14 g of PEG-6000 was added to 100 mL of 0.25 mol/L zinc sulfate heptahydrate. A sodium hydroxide solution (100 mL, 0.5 mol/L) was dripped very slowly over 24 hrs into the mixture under continuous stirring. Then, 100 mL of absolute ethanol was added as a co-surfactant. The resulting solution was stirred vigorously for 24 hrs. A white precipitate was formed, which was separated by centrifugation at 10.000 rpm for 5 min, washed with distilled water, and dried in a muffle furnace at 100 °C for 2 hrs. Then, it was ground into fine powder and finally calcined at 500 °C.

#### Sample 3

Ex-situ synthesis of ZnO-NPs using PEG-6000 as a stabilizer was performed by preparation of bare ZnO-NPs (Sample 1) followed by coating with PEG-6000 according to [14] with modification. Technically, the ZnO nanoparticles were dispersed in distilled water (1.5 w/v) at pH 10–11, that was controlled by NaOH. A solution of 10% PEG-6000 was then added wisely to the suspension of ZnO nanoparticles over 24 hrs. The mixture was stirred for a further 24 hrs. Then, the stabilized NPs were separated by centrifugation at 10.000 rpm for 5 min and washed 3–5 times to remove the excess polymer and dried at 50 °C.

#### Sample 4

The in situ prepared ZnO-NPs (Sample 2) were further coated with PEG-6000 by dispersing 15 mg of in situ prepared ZnO-NPs in 100 mL distilled water at pH 10–11 for 20 min. Then, 50 mL of PEG-6000 solution (10%) was added slowly drop-wise to the NP’s suspension and allowed to be maximally stirred for 24 hrs, and then sonicated for 1 hr. Parts of the PEG-6000 coated ZnO-NPs were separated by centrifugation at 14000 rpm for 5 min and characterized.

### Characterization of the prepared nanoparticles

#### Fourier transform infra-red (FTIR)

IR analysis was carried out on an FT-IR spectrometer (Nicolet 6700 FTIR, Shimadzu Co., Japan) to detect the interaction between ZnO-NPs and the stabilizer and characterize the interface of ZnO/PEG. A sample was compressed to a suitable KBr disc and scanned from 4000 to 400 cm^− 1^.

#### X-ray diffraction (XRD) analysis

The crystallographic structure of the obtained ZnO-NPs and the coated nanoparticles was examined by X-ray analysis. The average crystallite size (d) was determined from full width at half maximum (FWHM) of the diffraction peaks using Scherrer’s equation that is given as d = 0.9λ / (B. cos θ), where 0.9 is the Scherrer’s constant (0.7–1.7), λ is the wavelength of X-ray radiation beam, B is the integral width at half maximum and θ- glancing angle a given interference band [rad].

#### Transmission Electron microscopy (TEM)

A small amount of the PEG-coated in situ prepared ZnO-NPs powder was suspended in distilled water and sonicated for 20 hrs to ensure well dispersion. A drop of the previous solution was taken on a carbon-coated copper grid (400 mesh) for TEM imaging (JEOL JEM-100CX II Electron Microscope, Japan).

#### APEC strains

Ten APEC strains resistant to Florfenicol and Streptomycin; (O_146_, O_78_, O_2_, O_114_, O_163_, O_26_, O_91_, O_121_, O_127_, and O_124_) were isolated from broiler chickens in 2018 from Assiut, Egypt [15]. Bacteria were refreshed and propagated on Brain Heart Infusion (BHI) and Eosin Methylene Broth media (Oxoid Company, UK) at 37 °C for 24 hrs.

#### Assessing the antibacterial activity of the prepared ZnO-NPs samples

The 4 prepared ZnO-NPs samples were screened for their antibacterial activity against the APEC strains. Double-fold serial dilutions of each ZnO-NPs sample in a 0.4 mg/mL concentration with an adjusted bacterial concentration (5 × 10^6^ CFU/mL) were used to determine their Minimum Inhibitory Concentration (MIC) in BHI broth. A positive control contained only inoculated broth. The MIC endpoint is the lowermost concentration of the antibacterial, giving no visible growth.

#### Adjusting the minimum inhibitory concentration (MIC) of the in situ prepared PEG-6000 coated ZnO-NPs and its synergistic effect with Florfenicol and streptomycin

The standard “Tube macro-dilution” method according to [16], with some modifications was followed to assess the antimicrobial efficacy of the in situ prepared PEG-6000 coated ZnO-NPs (sample 4) by evaluating the visible bacterial growth on agar plates. Double-fold serial dilutions of the ZnO-NPs in concentrations 0.1, 0.2, and 0.4 mg/mL with each bacterial strain (5 × 10^6^ CFU/mL) were used to determine MIC in BHI broth. Also, double-fold serial dilutions of PEG-6000 coated ZnO-NPs in concentrations 0.1, 0.2 and 0.4 mg/mL combined with florfenicol and streptomycin (0.001 mg/mL form each antibiotic) were tested. The control contained only inoculated broth. All tubes were incubated for 18 hrs at 37 °C. Aliquots of 10 μL from all previously incubated tubes were seeded on EMB agar plates and incubated for 24 hrs at 37 °C.

#### Assessing the FIC index

The correlation between the PEG-6000 coated ZnO-NPs (sample 4) and the combined drugs were determined following the equation: FIC index = FIC_Ab_ + FIC_NP,_ Where, FIC_Ab_ = (MIC of antibiotic (Ab) in the presence of NPs) / (MIC of Ab alone) and FIC_NP_ = (MIC of NPs in the presence of Ab) */* (MIC of NPs alone). FICI value < 0.5 refers to synergism; FICI value between 0.5 and 1 means partial synergism and FICI = 1 means additive effect, while 2 < FICI< 4 indicates indifference and FICI > 4 refers to antagonism [17].

#### Determining the antimicrobial activity using the agar well diffusion method

A bacterial density of 5 × 10^6^ CFU/mL was poured evenly over the surface of EMB agar plates. Wells of 5 mm in diameter were made systematically in the agar plates using a sterile cork-borer. Then, each well was filled with 50 ϻL of the different PEG-6000 coated ZnO-NPs concentrations (0.1, 0.2, and 0.4 mg/mL) and PEG-6000 ZnO-NPs combined with florfenicol and streptomycin solutions at concentrations (0.001). The degree of sensitivity was estimated by measuring the visible and clear zone of inhibition. Results for florfenicol and streptomycin were analyzed according to [18], and PEG-6000 coated ZnO-NPs results were analyzed following the instructions of [19].

## Results

### Characterization of the prepared PEG-6000 coated ZnO-NPs

#### X-ray diffraction (XRD) analysis

**Fig. 1:**
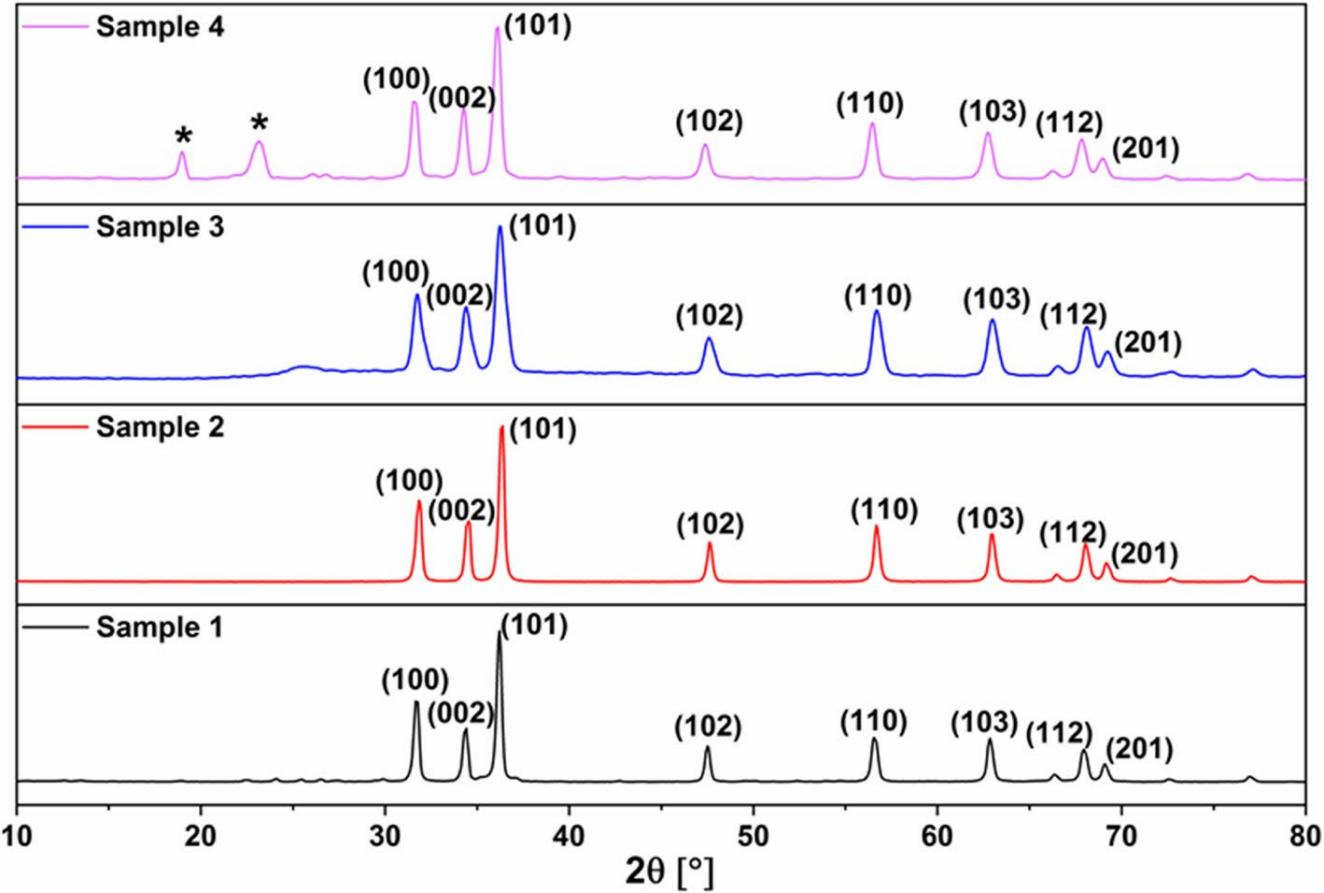
X-ray diffraction patterns of bare and coated ZnO-NPs. Sample 1: Bare ZnO-NPs showed typical peaks of the ZnO with a definite line broadening indicating the nano-scale with a mean crystal size of 25.8 nm. Sample 2: In situ prepared ZnO-NPs, using PEG-6000 as a surfactant and absolute ethanol as a co-surfactant, revealing the characteristic standard pattern of the ZnO-NPs with a crystal size of 28.7 nm. Sample 3: Ex-situ prepared ZnO-NPs using PEG 6000 as a stabilizer exhibiting a fine diffraction peak characteristic to the PEG (Arrow) and a definite line broadening indicate a smaller mean crystal size of 15.5 nm. Sample 4: In situ prepared ZnO-NPs coated with PEG-6000, after calcination, showing well-defined distinctive PEG diffraction peaks (stars) and mean particle size of 18 nm

XRD analysis has been carried out for all prepared samples, the powder patterns are shown in Fig. 1. For bare ZnO (sample 1), the powder pattern comprises reflections at 2θ = 31.70, 34.36, 36.21, 47.5, 56.57, 62.86, 67.92 and 69.1° that are typical for the wurtzite ZnO structure. No other reflections can be seen in the powder pattern indicating the phase purity of the obtained sample. Crystallite size estimation from line broadening using Scherrer’s equation revealed the nano-size feature; ZnO-NPs with a crystallite size of 25.8 nm were obtained.

For sample 2, the in situ prepared ZnO-NPs in the presence of PEG-6000 as a surfactant and absolute ethanol as a co-surfactant, the obtained powder pattern of which demonstrates also the characteristic reflections of ZnO with a crystallite size of 28.7 nm. No other reflections from the PEG-6000 can be seen in this sample.

**Fig. 2:**
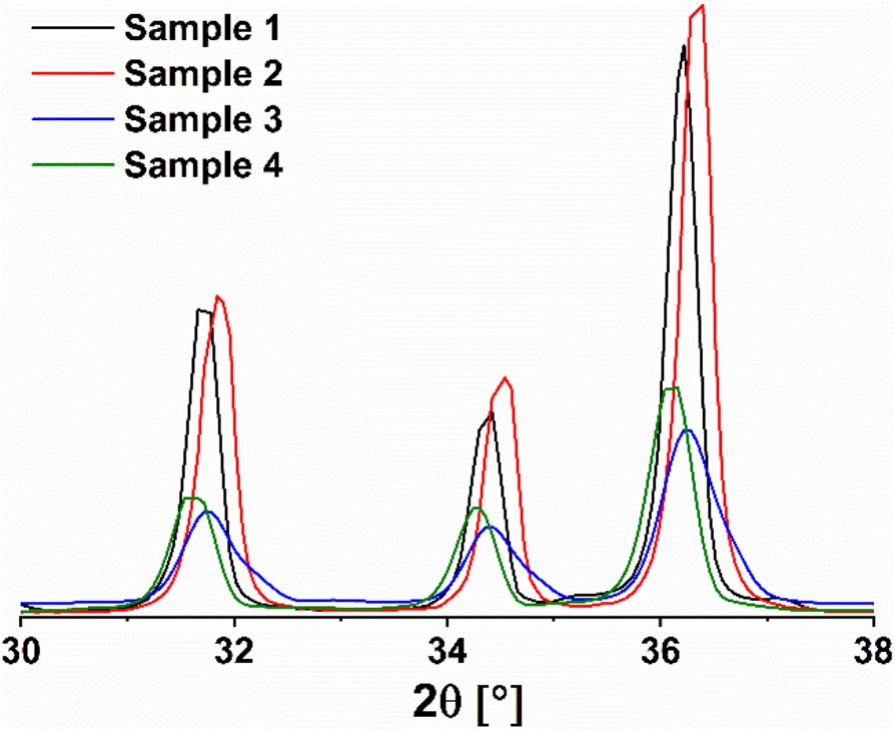
Magnification of XRD powder patterns of bare and coated ZnO-NPs

Although the ex-situ coated ZnO-NPs with PEG-6000 (sample 3) have displayed a powder pattern matched with that of the pristine wurtzite ZnO structure, some additional features can be observed. Peak broadening is well seen, reflecting the shrinkage of the crystallite size. The size drops from 25.8 to 15.5 nm. Ultimately, the powder pattern of sample 4 reveals the formation of ZnO-NPs. However, two additional reflections are apparent, at 2θ = 18.99 and 23.18°, that are distinctive for PEG-6000. The crystallite size of this sample drops from 28.7 to 18 nm. It is quite obvious from the magnification of XRD patterns shown in Fig. 2 that the coating process with PEG6000 performed either in-situ or ex-situ significantly affects both the peak shape and peak position. By coating, the peaks get less intense and broader, indicating the reduction of the crystallite size. Furthermore, the peaks undergo shifts in their positions reflecting structural changes.

#### Fourier transform infra-red (FTIR) analysis

**Fig. 3:**
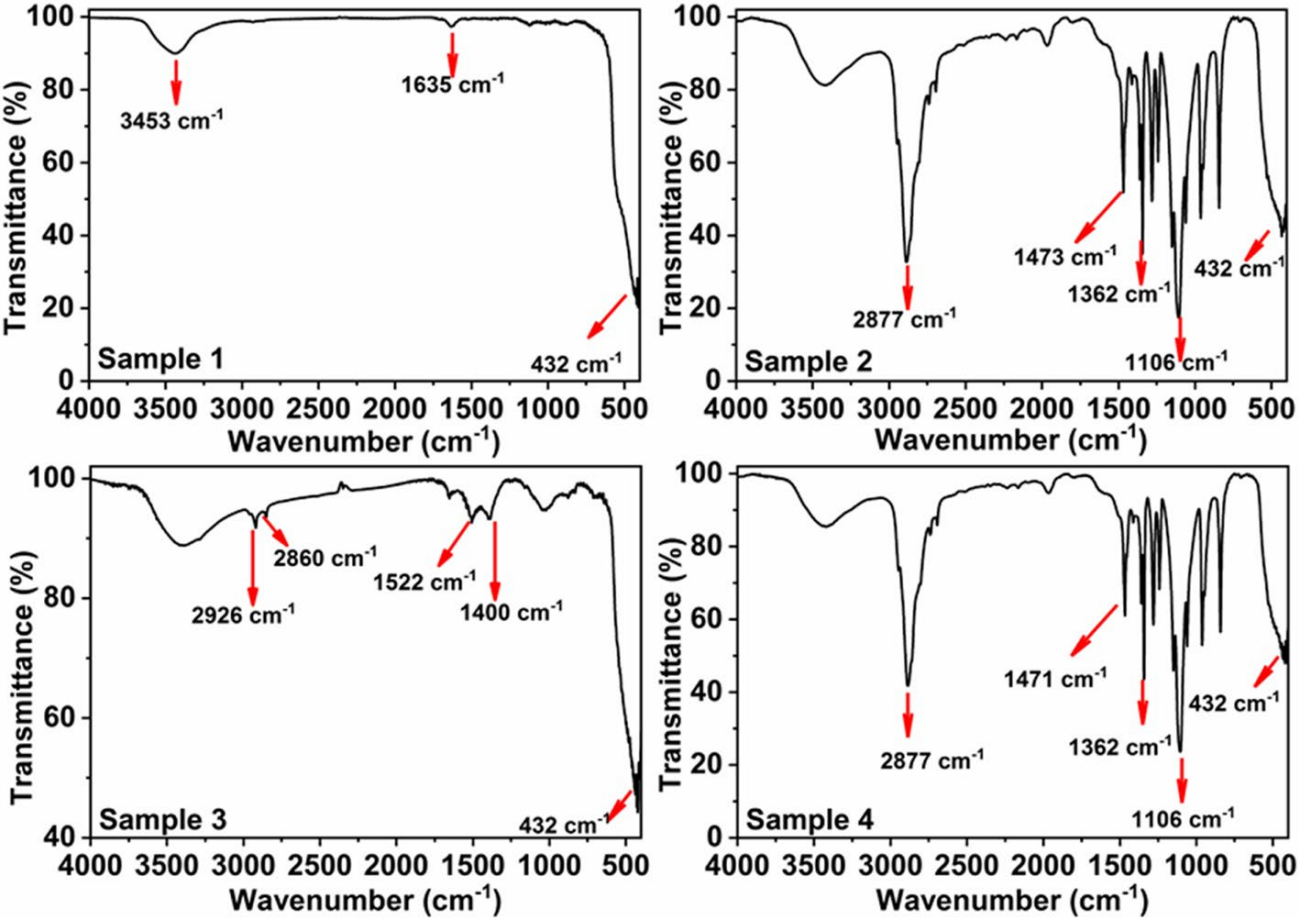
FT-IR spectra of bare and coated ZnO-NPs samples. Sample 1: Bare ZnO-NPs illustrate significant absorption peaks at ~ 3453, 1653, and 432 cm^−1^ (red arrows) that is characteristic to ZnO. Sample 2: In situ synthesized ZnO-NPs using PEG-6000 as a stabilizer and ethanol as co-surfactant exhibiting intense absorption peaks at ~ 1006, 1362, 1473, and 2877 cm^− 1^ (red arrows) confirming the existence of polymer molecules and metal oxides. Sample 3: Ex-situ synthesized ZnO-NPs using PEG-6000 as a stabilizer, showing absorption peaks of low intensity at ~ 1522, 2860, and 2926 cm^− 1^. Sample 4: The in situ prepared ZnO-NPs coated with PEG-6000 showing an assignment similar to sample 2

IR analysis was carried out for the bare and coated ZnO-NPs and the spectra are illustrated in Fig. 3. For all samples, an absorption peak seen at ~ 432 cm^− 1^ [20], which is characteristic to Zn-O stretching vibration. Moreover, two additional peaks are apparent at ~ 3453 and 1653 cm^− 1^ which are related to the stretching and bending modes of the adsorbed water molecules. For samples 2 and 4, IR spectra show intense absorption peaks at ~ 1006, 1362, 1473, and 2877 cm^− 1^ confirming the existence of polymer molecules covering ZnO nanoparticles. Sample 3 exhibited lower intensity peaks corresponding to PEG-6000 compared to samples 2 and 4.

#### Transmission Electron microscopy (TEM)

**Fig. 4:**
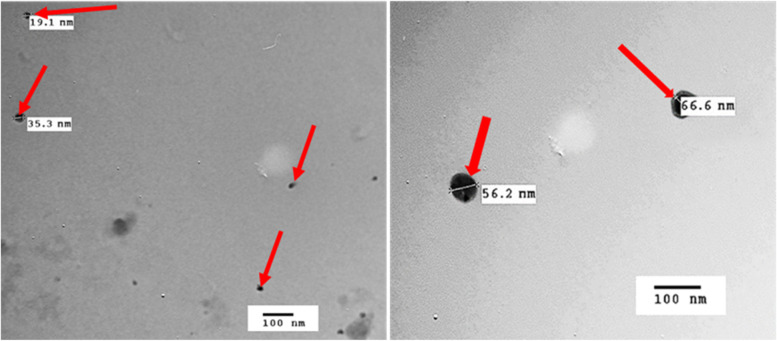
TEM photographs of PEG-6000-coated ZnO-NPs (sample 4) (red arrows) showing nearly spherical particle of good distribution and homogeneity within nano range

The morphological shape of PEG-6000 coated ZnO-NPs (sample 4) was investigated via transmission electron microscopy. TEM images depicted in Fig. 4 show that ZnO particles adopt a sphere-like morphology. The diameter of the nano-spheres was found to be in the range of 19–67 nm.

### The antibacterial activity of the prepared ZnO-NPs samples

The prepared ZnO-NPs samples displayed variable antibacterial activity in the broth media. The PEG-6000 coated in situ prepared ZnO-NPs (Sample 4) offered the best antibacterial effect (MIC = 0.1 mg/mL), with no observed bacterial growth in all concentrations, followed by the non-coated in situ prepared ZnO-NPs (Sample 2) of MIC =0.2 mg/mL.

The bare ZnO-NPs (Sample 1 and Sample 3) failed to inhibit the bacterial growth in all concentrations and the NPs were precipitated in the bottom.

### The antibacterial and synergistic effect of PEG-6000 coated ZnO-NPs against Florfenicol and streptomycin resistant APEC

#### The minimum inhibitory concentration (MIC) of PEG-6000 coated ZnO-NPs and PEG-6000 coated ZnO-NPs combined with Florfenicol and streptomycin

All tested APEC strains (100%) were sensitive to PEG-6000 coated ZnO-NPs (Sample 4) at a concentration of 0. 4 mg/mL, while only 30 and 20% of strains were sensitive to 0.2 mg/mL and 0.1 mg/mL, respectively (Table 1). The minimum inhibitory concentration was at 0.1 mg/mL in 50 and 30% sensitivity to PEG-6000 coated ZnO-NPs combined with florfenicol and streptomycin, respectively against APEC strains.

**Table 1:** The MIC values of PEG-6000 coated ZnO-NPs and PEG-6000 coated ZnO-NPs combined with florfenicol and streptomycin against APEC strains

***E.coli*** strains	MIC value (mg/mL)		
	PEG-6000 coated ZnO-NPs	PEG-6000 coated ZnO-NPs + Florfenicol	PEG-6000 coated ZnO-NPs + Streptomycin
**O146**	**0. 4**	**0. 4**	**R**
**O78**	**0. 4**	**0. 2**	**0. 2**
**O26**	**0. 2**	**0. 1**	**0. 2**
**O2**	**0. 2**	**0. 1**	**0. 1**
**O91**	**0. 2**	**0. 1**	**0. 2**
**O114**	**0. 4**	**0. 4**	**R**
**O121**	**0. 4**	**0. 1**	**0. 2**
**O127**	**0. 1**	**0. 1**	**0. 1**
**O124**	**0. 1**	**0. 2**	**0. 1**
**O163**	**0. 4**	**0. 2**	**0. 4**

#### Determination of fractional inhibitory concentration index (FICI)

Combining florfenicol with PEG-6000 coated ZnO-NPs revealed a synergistic effect in 30% of the tested APEC strains, a partial synergistic effect in 30% and an additive effect in 20%, while 20% of strains had an indifferent effect. Streptomycin in combination with PEG-6000 coated ZnO-NPs showed 10% synergism, 20% partial synergism, 50% additive effect and 20% not tested against the tested strains (Tables 2 and 3).

**Table 2:** FICI values of ZnO-NPs (sample 4) in combination with Florfincol and streptomycin

Strain	ZnO-NPs + Florfincol		ZnO-NPs + streptomycin	
	FICI value	Degree	FICI value	Degree
**O146**	1.08	AD	R	NT
**O78**	0.66	P.S	0.58	P.S
**O26**	0.5	S	1	AD
**O2**	0.5	S	0.5	S
**O91**	0.54	P.S	1	AD
**O114**	1.32	IN	R	NT
**O121**	0.29	S	0.66	P.S
**O127**	1	AD	1	AD
**O124**	2.04	IN	1.08	AD
**O163**	0.58	P.S	1	AD

*S* Synergy, *AD* Additive, *I* Indifference, *NT* Not tested

**Table 3:** Degree of interaction between florfenicol, streptomycin and PEG-6000 coated ZnO-NPs

Degree	PEG-6000 coated ZnO-NPs + Florfenicol	PEG-6000 coated ZnO-NPs + streptomycin
**Synergy**	30%	10%
**Partial synergy**	30%	20%
**Additive**	20%	50%
**Indifference**	20%	0%
**Antagonism**	0%	0%
**Not tested**	0%	20%

#### The antimicrobial activity by agar well diffusion method

**Fig. 5:**
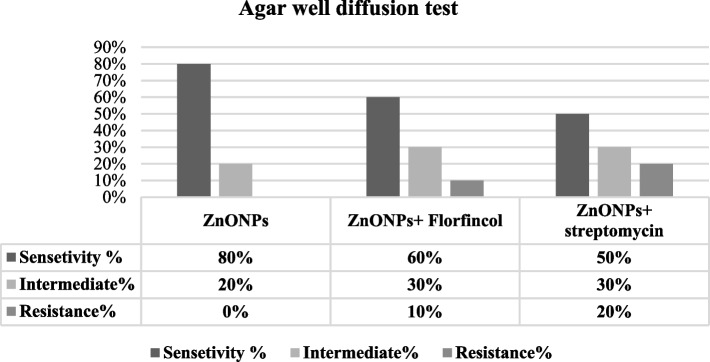
Sensitivity of APEC strains to PEG-6000 coated ZnO-NPs alone and PEG-6000 coated ZnO-NPs with florfincol and streptomycin according to well diffusion assay

All PEG-6000 coated ZnO-NPs concentrations inhibited the bacterial growth and created a maximum inhibitory zone of 20 mm at 0.4 mg/mL (80% sensitivity) and a minimum zone of 9 mm at 0.1 mg/mL. The synergistic effect between florfenicol and PEG-6000 coated ZnO-NPs revealed that 60% were highly sensitive, 30% were intermediate sensitive and 10% were resistant. Combining streptomycin with PEG-6000 coated ZnO-NPs showed that 50% of strains were sensitive, 30% were intermediately sensitive and 20% of strains were resistant. The inhibition zone of florfenicol started to appear in combination with 0.1 mg/mL PEG-6000 coated ZnO-NPs. Moreover, the maximum zone of inhibition reached 30 mm in combination with 0.4 mg/mL PEG-6000 coated ZnO-NPs. While the inhibition zone of streptomycin observed in combination with 0.2 mg/mL PEG-6000 coated ZnO-NPs and reached to the maximum zone (20 mm) with 0.4 mg/mL ZnO-NPs (Fig. 5).

## Discussion

APEC is responsible for major worldwide economic losses, including morbidity, mortality and the condemnation of poultry carcasses [21]. Because of the development of multidrug resistance among most APEC strains through alteration in the bacterial protein expression or mutations [22, 23], ZnO-NPs have attracted significant attention in medicine as an alternative or through regaining the activity of the available antimicrobials due to their small size, large surface area, low-cost, profuse nature, growth promotion, selective toxicity, antimicrobial and therapeutic activity, and safety as reported by the Food and Drug Administration [24, 25].

The XRD profile of sample 1 (bare ZnO-NPs) and sample 2 (*the* in situ prepared ZnO-NPs in the presence of PEG-6000 as a surfactant and absolute ethanol) showed the same distinct diffraction peaks typical for the wurtzite ZnO structure at 2θ = 31.70, 34.36, 36.21, 47.5, 56.57, 62.86, 67.92, and 69.1° with crystallite sizes of 25.8 nm and 28.7 nm, respectively, that came in accordance with the standard ZnO XRD pattern JCPDS No. 043–0002. The sharp characteristic peaks and absence of other peaks in the pattern of both samples reveal a desirable nano-crystalline structure, suggesting the high purity of the obtained ZnO. This result isn’t in congruence with [13], who concluded that using zinc sulfate as a precursor for bare ZnO-NPs yields particles with a size in the micrometer range.

The in situ addition of PEG-6000 (as a polymer) and ethanol during ZnO-NPs synthesis elicited a relatively larger crystallite size than the bare ZnO-NPs, which didn’t come in line with [13], who established that Polyvinyl Alcohol, as a polymer, addition during preparation of the ZnO-NPs decreases the particle size.

The XRD patterns of bare ZnO-NPs compared with that in situ prepared in the presence of PEG-6000 and ethanol indicates that the in situ addition of PEG-6000 during ZnO-NPs synthesis didn’t alter the XRD pattern of the ZnO-NPs and exhibited no additional reflections specific to PEG-6000.

Although being matched with the XRD pattern of the pristine wurtzite ZnO, the ex-situ coating of the bare ZnO-NPs with PEG-6000 (sample 3) exhibited additional features represented in peak broadening and the size dropped from 25.8 to 15.5 nm. While coating the in situ prepared ZnO-NPs (sample 4), the two additional reflection peaks at 2θ = 18.99 and 23.18°, which are distinctive ones for PEG-6000 with a dropped crystallite size of 28.7 to 18 nm. It is obvious that coating of either bare or in-situ synthesized ZnO-NPs using PEG-6000 significantly affects both the peak shape and peak position of the XRD pattern. The peaks get less intense, broader, and shift positions, indicating crystallite size reduction and structural changes. Conversely, to the authors who noticed that adsorption of PEG on the bare ZnO-NPs didn’t alter the crystalline construction of ZnO nanoparticles, and increasing the polymer molecular weight didn’t change the size of the coated NPs [14].

Regarding the Infrared (IR) spectra, the four samples presented absorption peaks characteristic to Zn-O stretching vibration (~ 432, 1635, and 3453 cm^− 1^) according to [20]. The FT-IR profile of sample 3 displayed lower intensity peaks corresponding to PEG-6000 compared to sample 2 (the in situ synthesized ZnO-NPs using PEG-6000 and ethanol) and sample 4 (the coated sample2). The more intense absorption peaks in sample 2 and their shift to low wave number reflect that the in situ use of PEG-6000 and ethanol during preparation of ZnO-NPs optimized the interaction and compatibility between the ZnO and PEG-6000. As well, the coating process was more efficient in case of sample 4 than sample 3 (ex situ coating of the bare ZnO-NPs).

The PEG-6000 coated in situ-prepared ZnO-NPs (Sample 4) represented nano-spherical morphology (∼19–67 nm) by TEM which indicate their capability to exhibit antibacterial activity. The smaller the particle size, the better biological activity through increasing interfacial area of reaction, the higher ROS release and the easier bacterial cell membrane penetration, hence inducing bacterial death more efficiently [24, 26]. In addition, ZnO-NPs release Zn2+ ions as a size-dependent phenomenon that may be responsible for the antimicrobial activity of ZnO-NPs causing bacterial cell wall rupture.

From the Minimum Inhibitory Concentration results in this study, the PEG-6000 coated in situ-prepared ZnO-NPs (Sample 4) elicited the best antibacterial effect (MIC = 0.1 mg/mL) followed by the (Sample 2) non-coated in situ-prepared ZnO-NPs (MIC = 0.2 mg/mL). The ex situ coated ZnO-NPs (Sample 3) mildly inhibited the bacterial growth, while the bare ZnO-NPs (Sample 1) completely failed to inhibit the bacterial growth in the broth media.

Failure of bare ZnO-NPs to induce antibacterial activity in the Minimum Inhibitory Concentration test may be due to their poor dispersibility and rapid precipitation in the broth (fluid) media, which accounts for the poor bioavailability, upon its application. Similarly, the scanning electron microscope (SEM) clarified that the bare ZnO-NPs are held together due to weak physical forces explaining particle agglomeration [13]. Moreover, suspending bare ZnO-NPs in the broth media, water molecules present, stimulated the formation of inter-particular Zn-O-Zn bonds and harsh particle agglomeration [10].

The in situ addition of PEG-6000 and ethanol as stabilizers (sample 2) lessened, somewhat, particles sticking together and allowed good dispersion in the broth media that displayed good antibacterial activity in the MIC. Likewise, the introduction of Poly Vinyl Alcohol as a surfactant during the synthesis of ZnO-NPs using zinc sulfate as a precursor sorted out the negative impact of particle agglomeration and particle separation [13].

Further coating of sample 2 with PEG-6000 (as a surface adsorbent) allowed better particle dispersion in the broth media and their protection against agglomeration or flocculation, making the NPs more attainable to the bacteria and improving the cellular incorporation of the nanoparticles and bacterial cell damage, consequently giving the maximal MIC value. This is attributed to the local solubilizing effect of PEG-6000 [27]. The polymer coating can effectively prevent water moiety in addition to modifying the particles’ surface through a combination of chemical and electrostatic interactions [14].

In this study, the antibacterial and synergistic activities of the optimized PEG-6000 coated in situ synthesized ZnO-NPs in the presence of PEG-6000 and ethanol (sample 4) were widely evaluated against 10 multidrug-resistant APEC strains. The minimum inhibitory concentration value of sample 4 was 0. 1 mg/mL.

A higher Minimum Inhibitory Concentration values of ZnO-NPs against *E. coli* were detected by [28] (0.15 mg/mL) and [29] who depended on ZnO nano-rods (0.250 mg/mL). It was noticed that the inhibition of *E. coli* requires high concentrations of non-coated ZnO NPs due to the cell wall lipopolysaccharides of APEC [6]*.*

Based on agar well diffusion test, all *E. coli* strains were sensitive to PEG coated ZnO-NPs. The inhibition zone started to appear at a concentration of 0.1 mg/mL with a minimum zone (9 mm) while the best result was achieved with a concentration of 0.4 mg/mL of ZnO-NPs, where the inhibition zone reached its maximum level (20 mm).

At concentrations of 0.1 mg/mL and 0.2 mg/mL the percentage of sensitivity was 20 and 40%, respectively. Similar results were observed by [30], who reported the inhibition zone of Zn-ONPs against *E.coli* at 13 mm. Also [31], who stated that the best antimicrobial activity of ZnO-NPs against *E. coli* was at 1 mg/mL, and the maximum zone of inhibition was at 13 mm. Authors stated that coating ZnO nano-crystals with polymers have antimicrobial effect may reach 100% inhibition of bacteria at low concentrations [25].

The interaction between florfincol and streptomycin with PEG-6000 coated ZnO NPs was observed as follows: 30, 10% synergism, 30 and 20%partial synergism, 20 and 50% additive effect, respectively. Drugs exhibited a 20% indifferent effect and there was no antagonistic effect using FICI. The maximum zone of inhibition to florfincol (30 mm) and streptomycin (20 mm) was detected in combination with 0.4 mg/mL ZnO-NPs by a well diffusion test.

The additive effect of ZnO-NPs with the other antimicrobial agents may be attributed to the difference in mode of action as when ZnO-NPs become in contact with the plasma membrane of a bacterial cell, leading to change in the cell permeability and ZnO NPs move to the cytoplasm and affect the normal functioning of cell resulting in the formation of zone of inhibition against the microbes [32]. Moreover, florfincol is proposed to disrupt bacterial protein synthesis and is generally considered to be bacteriostatic, while streptomycin works by blocking the ability of 30S ribosomal subunits to make proteins, which results in bacterial death, respectively. Furthermore, combinations of drugs are much more effective synergistically by improving the uptake or decreasing the elimination or degradation of another drug (e.g., by drug efflux pump blockage).

## Conclusion

Conclusively, keeping stable dispersion of ZnO-NPs is significantly important in the broth media to induce antibacterial activity. The in situ preparation of ZnO-NPs using PEG-6000 and ethanol enhanced their properties. The current findings refer to a perfect non-antibiotic antimicrobial alternative and an effective combination regimen against multidrug-resistant APEC *E. coli* in veterinary medicine. Combining coated ZnO-NPs with florfenicol and streptomycin enhanced their efficacy and could be successfully used in treating infection against APEC. It is worth further exploration for in vivo trials in poultry farms.

## Data Availability

The datasets generated during and/or analysed during the current study are available from the corresponding author on reasonable request.

## Abbreviations

APCE: Avian Pathogenic *E. coli*
FICI: Fractional inhibitory concentration index
MIC: Minimum inhibitory concentration
(PEG-6000): Polyethylene glycol 6000

## References

[1] McPeake S, Smyth J, Ball H (2005). Characterization of avian pathogenic Escherichia coli (APEC) associated with colisepticaemia compared to faecal isolates from healthy birds. Vet Microbiol.

[2] Prestinaci F, Pezzotti P, Pantosti A (2015). Antimicrobial resistance: a global multifaceted phenomenon, review. Pathogens Glob Health.

[3] Kim S, Woo JH, Jun SH, Moon DC, Lim S, Lee JC (2020). Synergy between Florfenicol and aminoglycosides against multidrug-resistant *Escherichia coli* isolates from livestock. Antibiotics.

[4] Lara HH, Ayala-Núñez NV, Turrent LDCI, Padilla CR (2010). Bactericidal effect of silver nanoparticles against multidrug-resistant bacteria. World J Microbiol Biotechnol.

[5] Matei A, Cernica I, Cadar O, Roman C, Schiopu V (2008). Synthesis and characterization of ZnO–polymer nanocomposites. Int J Mater Form.

[6] Reddy KM, Kevin F, Jason B, Cory H, Alex P (2007). Selective toxicity of zinc oxide nanoparticles to prokaryotic and eukaryotic systems. Appl Phys Lett.

[7] da Silva BL, Marina PA, Eloisa BM, João AO, Bruna G, RCL C-A, Pietro R, Leila Aparecida C (2021). Relationship between structure and antimicrobial activity of zinc oxide nanoparticles: an overview. Int J Nanomedicine.

[8] Bulcha B, Tesfaye JL, Anatol D, Shanmugam R, Dwarampudi LP, Nagaprasad N, Bhargavi V, Nirmal L, Krishnaraj R (2021). Synthesis of zinc oxide nanoparticles by hydrothermal methods and spectroscopic investigation of ultraviolet radiation protective properties. J Nanomater.

[9] Rodriguez-Paéz JE, Caballero AC, Villegas M, Moure C, Durán P, Fernández JF (2001). Controlled precipitation methods: formation mechanism of ZnO nanoparticles. J Eur Ceramic Soc.

[10] Yıldırım ÖA, Durucan C (2010). Synthesis of zinc oxide nanoparticles elaborated by microemulsion method. J Alloys Compd.

[11] Liufu S, Xiao H, Li Y (2004). Investigation of PEG adsorption on the surface of zinc oxide nanoparticles. Powder Technol.

[12] Tavakoli A, Ataei-Pirkooh A, Sadeghi GMM, Bokharaei-Salim F, Sahrapour P, Kiani SJ, Moghoofei M, Farahmand M, Javanmard D, Monavari SH (2018). Polyethylene glycol-coated zinc oxide nanoparticle: an efficient nanoweapon to fight against herpes simplex virus type 1. Nanomedicine (London).

[13] Mohan AC, Renjanadevi B (2016). Preparation of zinc oxide nanoparticles and its characterization using scanning electron microscopy (SEM) and X-ray diffraction (XRD). Procedia Technol.

[14] Nabiyouni G, Barati A, Saadat M (2011). Surface adsorption of polyethylene glycol and polyvinyl alcohol with variable molecular weights on zinc oxide nanoparticles. Iran J Chem Eng.

[15] Badry AM (2018). Detection and identification of antibacterial resistance genes of *E. coli* isolates from broiler chickens. M. Vet. Science. Thesis, Fac.Vet. Med. Assuit University.

[16] Parvekar P, Palaskar J, Metgud S, Maria R, Dutta S (2020). The minimum inhibitory concentration (MIC) and minimum bactericidal concentration (MBC) of silver nanoparticles against *Staphylococcus aureus*. Biomater Investig Dentistry.

[17] Smekalova M, Aragon V, Panacek A, Prucek R, Zboril R, Kvitek L (2016). Enhanced antibacterial effect of antibiotics in combination with silver nanoparticles against animal pathogens. Vet J.

[18] CLSI (2013). Clinical and laboratory standards institute (2013) performance standards for antimicrobial susceptibility testing, 23rd informational supplement. CLSI document M100-S23.

[19] Emami-Karvani Z, Pegah C (2011). Antibacterial activity of ZnO nanoparticle on grampositive and gram-negative bacteria. Afr J Microbiol Res.

[20] Said MI, Aly AA, El-Said AI, Abou-Taleb A (2020). Controlled synthesis of ZnO nanoparticles from a Zn (II) coordination polymer: structural characterization, optical properties and photocatalytic activity. Appl Organomet Chem.

[21] Ahmed AM, Shimamoto T (2013). Molecular characterization of multidrug resistant avian pathogenic Escherichia coli isolated from septicemic broilers. Int J Med Microbiol.

[22] La Ragione RM, Narbad A, Gasson M, Woodward MJ (2004). In vivo characterization of lactobacillus johnsonii FI9785 for use as a defined competitive exclusion agent against bacterial pathogens in poultry. Lett Appl Microbiol.

[23] Yang H, Chen S, White DG, Zhao S, McDermott P, Walker R, Meng J (2004). Characterization of multiple-antimicrobial-resistant Escherichia coli isolates from diseased chickens and swine in China. J Clin Microbiol.

[24] Wang C, Liu L, Zhang A, Xie P, Lu J, Zou X. Antibacterial effects of zinc oxide nanoparticles on Escherichia coli K88. Afr J Biotechnol. 2012.

[25] Ma L, Kohli M, Smith A (2014). Nanoparticles for combination drug therapy. NIH Public Access.

[26] Prasanna VL, Rajagopalan V (2016). Insight into the mechanism of antibacterial activity of ZnO: surface defects mediated reactive oxygen species even in the dark. ACS publications. Collection.

[27] Patil PH, Belgamwar VS, Patil PR, Surana SJ (2013). Enhancement of solubility and dissolution rate of poorly water soluble raloxifene using microwave induced fusion method. Braz J Pharm Sci.

[28] Kadiyala U, Turali-Emre ES, Bahng JH, Kotov NA, VanEpps JS (2018). Unexpected insights into antibacterial activity of zinc oxide nanoparticles against methicillin resistant Staphylococcus aureus (MRSA). Nanoscale.

[29] Taghizadeh SM, Lal N, Ebrahiminezhad A, Moeini F, Seifan M, Ghasemi Y, Berenjian A (2020). Green and economic fabrication of zinc oxide (ZnO) Nanorods as a broadband UV blocker and antimicrobial agent. Nanomaterials.

[30] Elkady MF, Shokry HH, Hafez EE, Fouad A (2015). (2015) construction of zinc oxide into different morphological structures to be utilized as antimicrobial agent against multidrug resistant Bacteria. Bioinorg Chem Appl.

[31] Farzana R, Iqra P, Shafaq F, Sumaira S, Zakia K, Hunaiza T, Husna M (2017). Antimicrobial behavior of zinc oxide nanoparticles and β-lactam antibiotics against pathogenic Bacteria. Arch Clin Microbiol.

[32] Sharma S, Kumar K, Thakur N, Chauhan S, Chauhan MS (2019). (2019): the effect of shape and size of ZnO nanoparticles on their antimicrobial and photocatalytic activities: a green approach. Bull Mater Sci.

